# A Review of the State of Dry Adhesives: Biomimetic Structures and the Alternative Designs They Inspire

**DOI:** 10.3390/mi8040125

**Published:** 2017-04-14

**Authors:** Jeffrey Eisenhaure, Seok Kim

**Affiliations:** Mechanical Science and Engineering, University of Illinois, Urbana-Champaign, IL 61801, USA; eisenhr2@illinois.edu

**Keywords:** dry adhesion, biomimetic, compliance, contact mechanics, shape memory polymer, fracture mechanics, review

## Abstract

Robust and inexpensive dry adhesives would have a multitude of potential applications, but replicating the impressive adhesive organs of many small animals has proved challenging. A substantial body of work has been produced in recent years which has illuminated the many mechanical processes influencing a dry adhesive interface. The especially potent footpads of the tokay gecko have inspired researchers to develop and examine an impressive and diverse collection of artificial fibrillar dry adhesives, though study of tree frogs and insects demonstrate that successful adhesive designs come in many forms. This review discusses the current theoretical understanding of dry adhesive mechanics, including the observations from biological systems and the lessons learned by recent attempts to mimic them. Attention is drawn in particular to the growing contingent of work exploring ideas which are complimentary to or an alternative for fibrillar designs. The fundamentals of compliance control form a basis for dry adhesives made of composite and “smart,” stimuli-responsive materials including shape memory polymers. An overview of fabrication and test techniques, with a sampling of performance results, is provided.

## 1. Introduction

The size of an animal influences their relationship with adhesion substantially as they navigate the world around them. As relatively large vertebrates, humans move primarily by using the weight of our bodies to produce friction between our feet and the ground below. Climbing smooth surface unaided is impossible, even for more agile species of ape or monkey which can nimbly climb trees by gripping trunks and branches around their circumference. An ant, by contrast, would find this method of climbing a grass blade or the face of your kitchen cabinet wholly unsuitable, having no trouble walking inverted on even the smoothest surface. The difference is, first and foremost, a matter of physical scale: the ant has far less mass in proportion to its surface area, and thus the surface forces acting upon it are vastly increased relative to its inertial forces. Many arthropods and smaller vertebrates have evolved specialized adhesive systems to take the fullest advantage of this principle, allowing them to deftly move about their natural environment with little regard for the shape or orientation of its surfaces.

The mechanisms by which the adhesive organs of these animals function remained largely elusive until a flurry of research in recent decades has begun to not only fully describe the morphology and properties of the organs, but also a wealth of experimental and theoretical evidence demonstrating the interaction of surface forces and kinetics to produce strong, controllable, and reliable adhesive contact [1,2,3,4,5,6]. The wealth of recent insights has helped to generate interest and hope among researchers that it is possible to develop an artificial dry adhesive system capable of mimicking the best that nature has to offer; a title frequently and deservedly awarded to the adhesive toe pads of the tokay gecko. Having a relatively large body mass for an animal relying on adhesive contact, the gecko is expected to require exceptional performance and indeed contemporary investigations have proven this to be the case [7]. Detailed investigation of the gecko toe pads reveals a complex hierarchical microstructure, formed of long and branched hairs terminated in sub-micron spatulae. These and other observations indicating that similar structures have independently evolved in many climbing animal species [8] have inspired the development and characterization of a great many artificial dry adhesives.

This paper reviews the principles governing the effective design and function of dry adhesive systems, drawing upon theory, observations of natural systems and experimental investigations of the artificial systems they have helped to inspire. There exist several reviews of dry adhesive research, published in recent years, which focus particularly on the design, fabrication, and applications for fibrillar adhesives [9,10,11,12,13,14,15,16]. During this time, a diverse set of alternative dry adhesives have been developed, typically intended to mitigate or circumvent the challenges inherent with the fabrication of fibrillar micro- and nanostructures. It is our intention with this work to provide a broad overview of the mechanics of dry adhesion, and by doing so to demonstrate that the performance of both fibrillar and alternative, non-fibrillar designs are determined by the same set of principles. We believe that viewing the challenge of developing an effective dry adhesive from this perspective will serve to enhance the rate of innovation within the field by helping researchers to consider the many possible combinations of structure and material. A brief summary of fabrication techniques and testing methods of artificial dry adhesive systems are provided in Section 3 and Section 4 respectively. Concluding remarks and the potential applications for the field of dry adhesives are provided in Section 5.

## 2. Design

The adhesive performance of two mating surfaces depends on many factors including their morphology, chemical makeup, cleanliness, loading conditions and environmental factors including temperature, light, humidity and the presence or absence of a surrounding fluid. The term performance, additionally, may describe a variety of characteristics of the adhesive pairing including failure stress for a particular set of loading conditions, work of adhesion, durability, and the ease of release in the case of reversible adhesives. In this section, we first consider the qualities which make for a desirable dry adhesive, and briefly discuss the fundamentals of how these qualities are achieved in practice. Observations from the natural world teach and reinforce the concepts of basic dry adhesion mechanics, guiding researchers first towards biomimetic surface patterning and fibrillar designs. Examples of alternative designs, generally intended to use the lessons of compliance control to create simpler but effective dry adhesives, are provided towards the end of the section.

### 2.1. Desirable Properties for Dry Adhesive Systems

Liquid adhesives and pressure sensitive adhesives (PSAs) are ubiquitous in modern society. The fundamental difference in function between the more common artificial liquid and PSAs, and a typical dry adhesive, is the reusable and releasable nature of the dry adhesive. While a liquid adhesive or a PSA cures or flows to bond two surfaces together permanently (or at the very least, cannot be reused with the same performance should the surfaces be later separated), a dry adhesive is intended to create a nondestructive temporary bond which may be undone and repeated many times without prohibitively degrading its adhesive performance between bond cycles.

#### 2.1.1. Strong and Reversible Adhesion

A useful dry adhesive must, first and foremost, be capable of generating a bond of adequate strength to its adherend for its intended application, in what we may refer to as the dry adhesive’s “adhesion on” configuration. The strength of an adhesive bond is typically measured either in terms of its load bearing capacity at failure, or in terms of the energy dissipated during the separation of adhesive and adherend. This measured “maximum” adhesive strength of a dry adhesive bond will vary depending on many factors regarding both adhesive and adherend, but is typically several orders of magnitude below that of a comparably sized “wet” adhesive bond, which may support loads well in excess of 1 kN/cm^2^ [17]. Nonetheless, dry adhesives are capable of generating adequate strength for low and moderate load situations, particularly when bond area may be increased.

The weaker bond of a dry adhesive is the price paid for its reusability, and corresponding ability to detach non-destructively from its adherend. Though less strong than permanent bond methods, detachment through a dry adhesive’s primary loading path is usually prohibitively difficult. Therefore, the design of a useful dry adhesive will include a method of facile detachment; the detachment method typically involves altering the load path and failure mode of the interface, or in the case of more recent dry adhesives created with “smart”, stimuli-responsive materials, the detachment is facilitated by a stimulus-assisted change in the adhesive’s material properties or morphology. When loaded or otherwise prepared in this way for easy detachment, we say the dry adhesive is in its “adhesion off” configuration, and the measured resistance to detachment may be called the adhesive’s “minimum” adhesion. As with the maximum adhesion, the minimum adhesion achieved by a dry adhesive varies greatly with the design of the adhesive and the properties of the adherend.

The ratio of maximum to minimum adhesion will be referred to as the adhesive’s “reversibility”, and is an important metric for dry adhesive design, particularly for very small-scale applications where surface forces dominate inertial forces, and for applications where speed and efficiency are of significant importance such as the motility of certain animals and robots. A climbing insect possessing footpads with poor adhesive reversibility, for example, would find locomotion to be challenging: either because the maximum adhesion would be too poor to allow adequate traction, or the minimum adhesion too great to allow for timely and versatile detachment of individual foot pads.

#### 2.1.2. High Adhesion to Preload Ratio

It is generally desirable to minimize the necessary compressive force between adhesive and adherend necessary to create a strong bond. The ratio of an adhesive’s maximum adhesive force to the corresponding force, or preload, required to form the bond, is herein referred to as its adhesion to preload ratio. Though often given little attention in current artificial dry adhesive research, it is of great importance for most practical applications. A lizard attempting to climb a wall would tire quickly having to exert itself against the wall with each step. Likewise, a person wishing to hang a television on their wall with a dry adhesive mount would rather not have to apply a force comparable with their television’s weight against the wall, and a manufacturer utilizing dry adhesives to manipulate delicate structures will value a low-preload process both to protect their products and boost efficiency. In the ideal case, a dry adhesive will generate its full adhesive strength passively upon contact with its adherend.

#### 2.1.3. Durability

A reusable dry adhesive must have a durable adhesive surface which is resistant to damage and fouling, each of which can degrade performance substantially. An adhesive surface often experiences significant cyclic stress and corresponding strain during a loading and unloading cycle, often in shifting and perhaps unpredictable directions requiring that the materials and surface structuring work together to form a mechanically robust surface. Fouling by means of particulate contamination is a significant concern for any reusable adhesive. It must be expected that the surface of any adherend will contain some particle debris which may be transferred to the adhesive. The challenge is to design the adhesive such that it will adhere to the adherend, but resist collecting particles, or having collected the particles the surface will remove them within a few attach and detach cycles or by flowing liquid or capillary action, a characteristic and process often referred to as “self-cleaning” [18,19,20,21,22]. The issue of surface fouling is particularly challenging and restricts the use of most current artificial dry adhesives to use with very clean adherends, lest their performance undergo substantial degradation.

### 2.2. Fundamental Concepts for Creating a Dry Adhesive System

All dry adhesive systems must obey the same set of physics. The performance requirements of each system dictate the particulars of its design. This section summarizes the current understanding of the relevant design principles identified by researchers for reversible dry attachment to surfaces with varied surface chemistry and morphology.

#### 2.2.1. Attractive Forces

An attractive force between two surfaces may be effected through numerous mechanisms including mechanical interlocking; long-range electrostatic interactions; short-range electrostatic interactions (van der Waals forces, hydrogen bonds); molecular bonding (ionic, covalent, metallic); magnetic forces; capillary forces; and the Casimir–Polder force. Though among the weakest of these in terms of the maximum potential attractive force, the van der Waals forces are most frequently the dominant contributor to the performance of dry adhesive systems [1,23,24,25,26]. The van der Waals forces arise from very short range (3–7 Å) [27] interactions between permanent and induced molecular dipoles, comprised of the Keesom, Debye, and London dispersion forces [28]. The forces arise spontaneously when two surfaces are brought into contact and are ubiquitous, occurring with varying extent for all neighboring molecules and mostly independent of other environmental factors. This means that a dry adhesive relying on van der Waals forces should be expected to perform similarly with chemically diverse adherends and with little intrinsic effect from temperature, pressure, humidity or external electromagnetic fields [29]. The relative weakness of the bonds may, in fact, be considered an advantage for a reversible dry adhesive because it allows for faster and more efficient detachment with virtually non-existent surface damage or fouling to either adhesive or adherend, each maintaining surface mechanical integrity through their much stronger covalent or ionic bonds.

The bond between two materials is frequently thought of and described in terms of surface energies. It is energetically favorable for solid materials to minimize their free surface area due to the summation of internal short range molecular forces, and the energy required to create the free surface is denoted *γ* (units of N/m). Two free surfaces, designated *a* and *b*, brought into contact will then require work to separate, referred to as their thermodynamic work of adhesion *W_ab_*. The work of adhesion between surfaces *a* and *b* is related to their individual surface energies, and interfacial energy *γ_ab_*, as [30]:

(1) $$W_{ab}=\gamma_{a}+\gamma_{b}-\gamma_{ab}$$

The value of *γ_ab_* is possible to estimate using the work of Girifalco and Good as [31]:

(2) $$\gamma_{ab}=\gamma_{a}+\gamma_{b}-2\sqrt{\gamma_{a}\gamma_{b}}$$

Combining Equations (1) and (2) gives the result that $W_{ab}\approx 2\sqrt{\gamma_{a}\gamma_{b}}$, which for the case of cleaving a single uniform material to create two new surfaces gives the sensible result that W≈2γ. In the case of strong covalent or ionic bonds, it may be expected that 2*γ* ≈ 2 N/m [32]. For van der Waals forces, those which dominate in the case of dry adhesion, a more typical value is 2*γ* ≈ 0.05 N/m [33]. A small number of artificial dry adhesive systems have been developed to enhance performance using long-range electrostatic forces with high voltage power sources [34,35,36,37,38] as a supplement to van der Waals forces. Capillary forces are more often a significant or suspected contributor to adhesion, particularly for biological systems adapted to function in wet or humid environments [1,39,40,41,42,43,44]. However, cases where the presence of a liquid layer and meniscus are confirmed to be substantially important for the functioning of a reusable adhesive surface are still somewhat uncommon.

#### 2.2.2. Controlling Elastic Energy

The short range of van der Waals interactions requires molecules to be essentially “touching”, in the molecular sense to generate an appreciable adhesive force between them. The sum of the van der Waals attractive force between two objects will scale with the intimate contact area between them, as proportionally more molecules are brought close enough to attract each other. Increasing this sub-nanometer contact is a basic goal for the design of a dry adhesive system when more adhesive strength is required, and adhesive strengths are frequently reported on a per-area basis with the rough assumption that adhesive strength will scale linearly with adhesive area. Classical contact mechanics, as developed by Heinrich Hertz, describes the contact between a spherical elastic body and an elastic half-space in terms of the compressive force between them *F*, the sphere radius *R*, contact radius *a*, and the system’s effective modulus E*, which is a function of each material’s elastic modulus and Poisson ratio:

(3) $$a^{3}=3FR/4E^{*}$$

The Hertzian model accounts for the elastic restoring forces within the bodies to resist a compressive load, however the attractive surface forces are disregarded. An expanded model of elastic contact accounting for surface forces was put forth by Johnson, Kendall, and Roberts, and is consequently referred to as the JKR model of elastic contact [23]. The inclusion of the thermodynamic work of adhesion *γ* modifies the Hertzian equation as follows:

(4) $$a^{3}=\frac{3R}{4E^{*}}\left(F+3\gamma \pi R+\sqrt{6\gamma \pi RF+{\left(3\gamma \pi R\right)}^{2}}\right)$$

**Figure 1:**
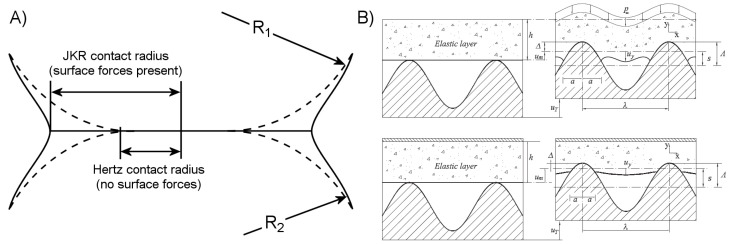
(**A**) The contact between two convex elastic bodies of radii *R*_1_ and *R*_2_ under a compressive normal load in the presence and absence of surface forces. (**B**) Elastic layers in contact with a rough or wavy surface assuming the layer is free (top) or confined (bottom) (reproduced with permission from [47]; published by Elsevier, 2016).

Attractive surface forces expand the contact area, as depicted in Figure 1A. The physics described by this relation has great significance for adhesive performance since real surfaces are seldom atomistically flat, but are instead rough, covered with asperities which inhibit contact. The asperities are analogous to the elastic sphere compressed against the opposing half-space. For a given work of adhesion, typically in the range of 50 mJ/m^2^, it is apparent that soft materials are necessary for conformal contact to occur. The reduced restoring force of the softer interface allows attractive surface forces to dominate, and the materials to deform and “flow” around asperities to increase surface area and therefore adhesion. In the common case of a rigid adherend, it is the duty of the adhesive to undergo the majority of this deformation (see Figure 1B), and an adhesive which performs this task adequately is referred to as being sticky, or in more technical terms as having “tack” [45,46].

A tacky adhesive is one which, immediately upon contacting an adherend, will require a significant force to separate. A common rule of thumb for pressure sensitive adhesives (PSAs) is the Dahlquist Criterion, suggested by Carl Dahlquist in 1969, which roughly states that the elastic modulus must be below 3 × 10^5^ Pa to exhibit tack against common surfaces. The exact value depends upon the nature and roughness of both the adhesive and the adherend. For a somewhat idealized surface modeled as a regular series of bumps with radius *R* and height 2*h*, the critical elastic modulus *E_c_* for a material to exhibit tack can be calculated as [48]:

(5) $$E_{c}=W\sqrt{R/h^{3}}$$

where *W* is the thermodynamic work of adhesion. In certain cases where this criterion is not strictly met, conformal contact may still be achieved through the application of compressive force, or preload, to unite the two surfaces. Enhancing the compliance of an adhesive is not entirely “free” in terms of performance, however. Adhesive interfaces are strongest when loaded evenly, i.e., when the stress is well distributed. Compliant adhesive structures are less able to resist deformation, and therefore more susceptible to concentrated stresses [49], for example when peeled. Excessive strains reduce mechanical durability, and the tacky surface of a low-modulus adhesive is more susceptible to fouling through particle contamination.

**Figure 2:**
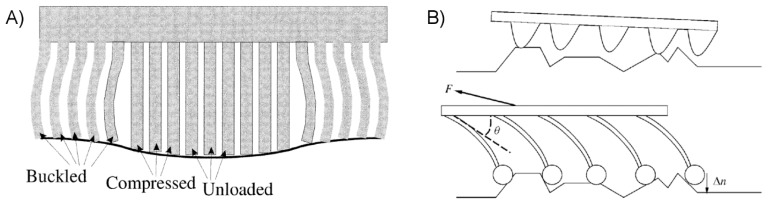
(**A**) Fibrillar surface loaded in compression against a rough or uneven surface (reproduced with permission from [52]; published by Oxford Academic, 2002). (**B**) Comparison between the conformal ability of low aspect ratio nanobumps and longer, more flexible nanohairs (reproduced with permission from [53]; published by Taylor and Francis, 2003).

A possible solution to the challenges of adhering to rough surfaces while minimizing the negative impact of excessively low modulus comes in the way of surface structuring. Researchers have long been aware that many small climbing animals and insects have evolved adhesive organs with complex morphology. In particular, their surfaces are frequently coated with relatively slender fibers or hairs, broadly referred to as fibrillar surfaces. Recent research has shed light on the mechanisms by which fibrillar structures enhance adhesion, and many attempts of artificial mimicry have been made. A well-established benefit of fibrillar surfaces is their enhanced surface compliance and ability to conform to a rough adherend even when composed of a relatively high-modulus material. Individual micro- and nano-scale fibers may bend and buckle, reaching past adherend asperities to contact the microscopic valleys between, as shown in Figure 2A. Fibers which are adequately long and flexible can form a similar level of contact quality for smooth and rough surfaces alike (Figure 2B). The JKR and similar models for adhesive contact additionally indicate that even for a flat and smooth adherend, a dense array of small contact points will provide better adhesion than a few larger contact points due to the reduction in the elastic deformation necessary to create a given contact area. The principle of increasing the number of contact points to enhance dry adhesive performance is well-established and referred to as contact splitting [50], though the extent to which contact splitting directly enhances fibrillar adhesion is not entirely clear [51].

**Figure 3:**
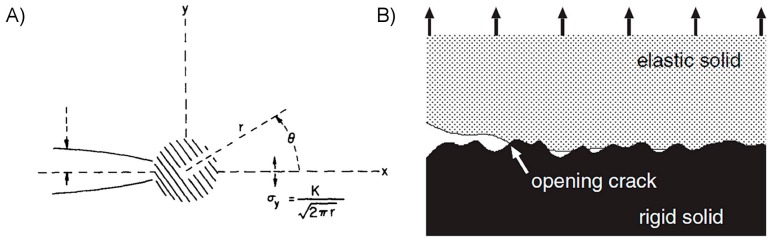
(**A**) Schematic view of the leading edge of a crack in an elastic body (reproduced with permission from [56]; published by Elsevier, 1968). (**B**) An opening of a crack in an adhesive interface during pull-off (reproduced with permission from [57]; published by Elsevier, 1968).

Regardless of the method of forming an adhesive interface, a designer is naturally interested in understanding how its morphology affects its strength and performance during use. Fracture mechanics consists of a set of methods to predict the onset and propagation of cracks, considering both solid mechanics and surface forces [32,54,55]. Initially developed by Griffith to investigate brittle failure in homogeneous materials, and later expanded by Irwin and then Rice to include the effects of plastic dissipation as depicted in Figure 3A, it may easily be adapted to describe many adhesive interfaces. A typical linear analysis will first assume a material or adhesive interface possesses a pre-existing crack. Crack advance is determined by an energy balance comparing, for an infinitesimal advance of the crack, the energy release rate to the critical energy necessary to separate the surfaces, or “work of adhesion.” The energy release rate is the summation of the rate of change of internal strain energy and the work done by tractions on the system boundaries. If the energy released exceeds the energy required to create the new surfaces, then the crack will advance. Irwin’s treatment developed the concept of the crack stress intensity factor, *K*, which is calculated separately for each of three modes of failure as a function of the system’s geometry and loading conditions: Mode I tensile opening, Mode II in-plane shear, and Mode III out-of-plane shear. The values of *K* are calculated from the stress field near to the crack tip for Mode *i*:

(6) $$K_{i}=\underset{r\to 0}{\lim}\sqrt{2\pi r}\sigma_{jk}\left(r,0\right)$$

where the subscripts *j* and *k* are placeholders describing relevant stress plane. The solution of Equation (6) gives a result for Mode I loading typically of the form:

(7) $$K_{I}=C\sigma \sqrt{\pi a}$$

where *C* is a constant particular to the crack geometry and loading conditions, *a* is the crack length, and *σ* is the applied tensile stress far from the crack. It is evident that longer cracks significantly increase the stress intensity factor, and therefore decrease the expected failure strength of the material or interface. Modes I and II are most frequently dominant for adhesive interfaces, and are related to the energy release rate *G* as follows (plane strain):

(8) $$G_{i}=\frac{\left(1-\nu^{2}\right)}{E}K_{i}^{2}$$

where the material’s elastic modulus *E* and Poisson’s ratio *ν* are included. Using work of adhesion *W_a_* as the failure criterion, where failure is predicted when *W_a_* < *G*, it is apparent from Equation (8) that a greater elastic modulus should be expected to increase the adhesive’s strength assuming that it is capable of forming intimate conformal contact to its adherend.

The approach above can provide useful predictive power for the strength of adhesive interfaces which meet the assumptions inherent in linear fracture mechanics. Its application can be less meaningful for non-ideal interfaces where an interface cannot be assumed continuous and homogeneous, such as the one shown in Figure 3B. A more general and simple framework to guide the mechanical design of dry adhesives is desirable. One such relation has been developed recently by Bartlett et al. using a simplified energy balance for an elastic body of arbitrary shape, assuming unstable interfacial separation will occur at a critical force *F_c_* [58]. The critical force is proportional to interfacial surface area *A*, compliance *C*, and interfacial critical energy release rate *G_c_* as:

(9) $$F_{c}\;\sim \;\sqrt{G_{c}}\sqrt{A/C}$$

The researchers experimentally tested their own composite adhesives comprising a variation of four orders of magnitude of $\sqrt{A/C}$, demonstrating general agreement with Equation (9) for bonds to a smooth adherend. Additional evidence indicating the broad applicability of this simple relation has been provided by subsequent experimental work [59,60,61], including an investigation for the relation’s applicability for rough adherends where it was concluded that even subtle surface roughness dramatically impacts the expected adhesion due to elastic restoring forces, particularly for rigid adhesives [62]. The work highlights the importance of compliance control in a dry adhesive system, and reinforces that superior performance may be expected for an adhesive capable of displaying large compliance when conforming to an adherend, while remaining inextensible when loaded.

#### 2.2.3. Enhancing Work of Adhesion through Energy Dissipation and Absorption

The work of adhesion for a strong adhesive is on the order of 100–1000 J/m^2^. This value is notably much larger than the thermodynamic work of adhesion for typical material pairs (~100 mJ/m^2^) and even much greater than ionic or covalent bond energies (~2 J/m^2^). The source of this discrepancy, and methods to enhance it, have been the subject of considerable research.

**Figure 4:**
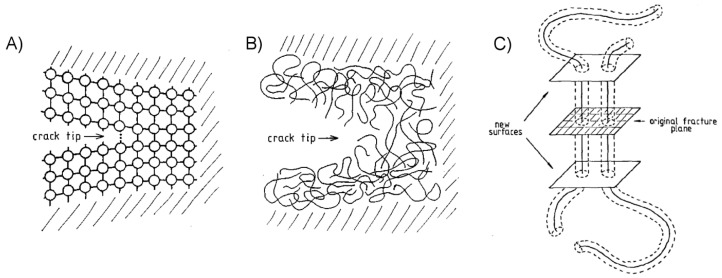
(**A**) A crack in a brittle atomic crystal, and (**B**) a crack in a brittle polymer (reproduced with permission from [73]; published by the American Institute of Physics, 1999). (**C**) Schematic representation of the “pull-out” process occurring during crack propagation within a polymer (reproduced with permission from [68]; published by Springer, 1985).

It has long been observed that the cohesive bond strength, or tear resistance, of rubbers is significantly greater than what should be predicted by the energy of their covalent bonds alone [63,64]. Rubber and other polymeric materials are formed of covalently-bonded molecular chains which can move relative to each other under load, and in the process dissipate energy. The materials are described as viscoelastic, meaning they exhibit both reversible elastic and irreversible viscous responses when undergoing deformation. The molecular arrangement of a crack forming in a crystalline material is compared with that of a polymer in Figure 4, showing the less-ordered arrangement of polymer chains bridging the crack front. Viscous energy dissipation and crack-tip phenomena (crazing, blunting) have been identified as the primary causes of the impressive tear resistance in many soft polymers [33,65,66,67]. Crack blunting is significant when a polymer’s cohesive strength matches or exceeds its elastic modulus, and is the result of large nonlinear elastic deformations which act to reduce the stress at the crack tip [33]. Lake and Thomas posited that for the crack to advance, each polymer chain bridging the crack path must be broken, though for a single bond to be broken the entire chain must be stretched to near its breaking point [63]. The energy required to stretch the chain is then dissipated, rather than elastically returned to the bulk material, thus enhancing the material’s fracture strength. This is also thought to occur in the case where, instead of breaking, a polymer chain bridging the original fracture plane slides or “pulls-out” from the opposing side, as shown in Figure 4C [68]. Bulk viscoelastic processes dissipate energy throughout the material as it is loaded, the effect being especially pronounced for a polymeric material near its glass transition temperature and with relatively little molecular crosslinking [69,70]. These internal molecular processes likewise serve to enhance the work of adhesion for an adhesive interface between a polymer and its adherend [64,71,72].

**Figure 5:**
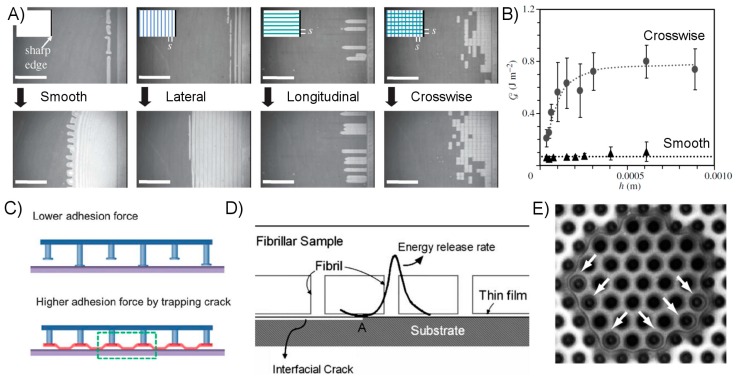
(**A**) The effect of discontinuities on interfacial crack propagation using differently incised polydimethylsiloxane (PDMS) films, and (**B**) the relationship between fracture energy and film thickness for smooth and crosswise incised films (reproduced with permission from [74]; published by Royal Society, 2005). (**C**) Illustrations of crack trapping for a film-terminated fibrillar adhesive pulled normal to the adherend (reproduced with permission from [81]; published by Royal Society of Chemistry, 2013), and (**D**) showing how the energy release rate varies depending upon the position of the crack front (reproduced with permission from [80]; published by Royal Society of Chemistry, 2008). (**E**) An optical image of a film-terminated fibrillar adhesive delaminating from an adherend, showing interfacial cavitation under several fibers (reproduced with permission from [76]; published by the National Academy of Sciences, 2007).

Researchers have additionally identified certain forms of surface geometry or structuring which enhance energy dissipation, and therefore adhesive strength. Chief among these are the fibrillar structures frequently found on climbing animals in nature, which have been suggested to dissipate energy in a process analogous to the molecular stretching of polymeric chains; an individual fiber will bend and elongate until its contact with the adherend breaks, at which point the energy invested in deforming the fiber is dissipated inelastically rather than returned to the bulk material [52,72]. The smaller and more numerous contact points of a fibrillar adhesive enjoy the additional benefit of minimizing the crack length at each interface [6]. The benefit of this from a fracture mechanics standpoint is apparent from Equation (7). It has additionally been shown that inhomogeneous or partitioned surfaces can enhance the adhesive performance of thin films, in particular the interfacial fracture toughness [74,75]. The incisions create many internal cracks which act as defects to disrupt and deflect the continuous crack propagation which occurs in a smooth film, as depicted in Figure 5A. The thickness of the film was found to play a role, with thicker films producing greater energy dissipation as the taller segments are stretched further prior to delamination, and thus absorb and dissipate more elastic energy (Figure 5B). Engineering an adhesive surface such that energetic barriers to crack propagation exist is frequently referred to as “crack trapping”, and many researchers have used it to their advantage in the creation of film-terminated fibrillar dry adhesive surfaces (Figure 5C–E) [76,77,78,79,80,81]. The fibrillar structure supporting the smooth film surface creates “trap” regions between the fibers where the film absorbs energy which would otherwise be applied toward crack propagation. Further separation may only occur as the stress beneath the fibers increase enough to spontaneously form new cracks, a circumstance requiring substantially more force and energy than for a corresponding flat and unstructured surface. Figure 5E depicts the situation in which separation between adhesive and adherend occurs spontaneously below fibers rather than by continuous crack propagation between fibers.

### 2.3. Strategies for Creating a Successful Dry Adhesive

#### 2.3.1. Observations of Natural Systems

It is common knowledge that insects, many frogs, and small lizards are impressively skilled at climbing on all manner of surfaces and in all orientations. These animals possess adhesive pads on their toes or legs which are capable of adhering to the surfaces in their natural environment, and just as importantly, are capable of detaching with relative ease. The sequential attachment, loading, and detachment are essential to effect locomotion. Modern investigation has determined that although no two species may possess exactly the same attachment organs, there are substantial similarities in form and function even between evolutionarily very distinct animals. The form of adhesive pads can generally be described as either smooth or hairy.

**Figure 6:**
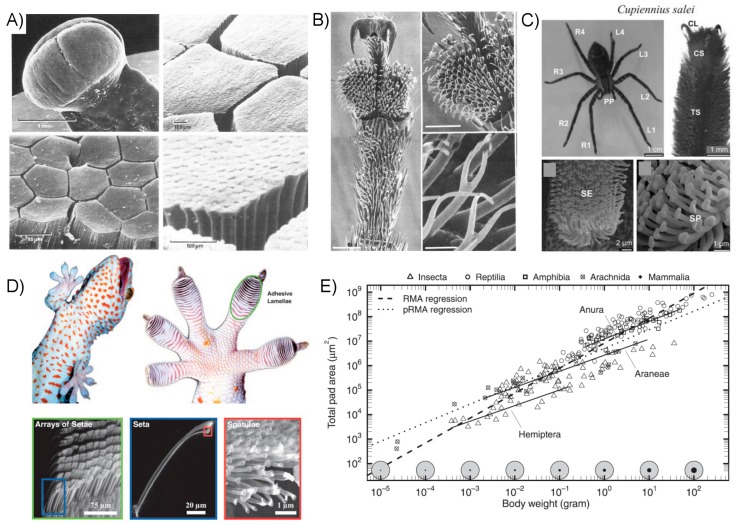
(**A**) Images of the toe pad of a frog, showing individual epidermal cells and mucous glands (reproduced with permission from [93]; published originally by Wiley, 1980, copyright Oxford Academic). (**B**) Scanning electron microscope images of the tarsus of the bruchus atomarius beetle (reproduced with permission from [94]; published originally by Wiley, 1980, copyright Oxford Academic). (**C**) Images of a spider’s adhesive organs (reproduced with permission from [95]; published by Springer, 2006). (**D**) The structural hierarchy of the gecko adhesive system (reproduced with permission from [24]; published by Oxford Academic, 2002). (**E**) The relationship between body mass and pad area for a variety of animal taxa (reproduced with permission from [96]; published by the National Academy of Sciences, 2016).

Smooth, when referring to adhesive organs, is a rather loosely used term because the organs often have some low-aspect ratio micro-scale structuring to them. Animals possessing smooth pads include crickets [2,82], ants [3,42], bees [3,83], cockroaches [84,85,86], stick insects [87], aphids [4,88], and tree frogs [89,90,91,92]. It is frequently unclear for biological systems to what extent the adhesion is truly “dry”, particularly in the case of smooth attachment pads. Many insects secrete emulsions such that the pads are continually coated in a wet or oily substance. Study of stick insects has determined that a substantial portion, perhaps the majority, of their adhesive strength from emulsions with non-Newtonion properties to resist shear forces [87]. At least some species of aphids, which spend a great deal of their time walking along the wet and humid surface of leaves, rely primarily on surface tension of expelled fluid on their adhesive pads to scale smooth surfaces [4]. When scaling rough surfaces, they may eschew use of their adhesive pads altogether in favor of clinging with claws [88], a trait shared by insects of the Hymenoptera order including ants and bees [3]. The adhesive mechanisms of tree frogs are still not fully understood by researchers, though their hexagonally-patterned toe pads are wetted with watery mucus (Figure 6A). Theoretical and experimental research has suggested contributions to their clinging ability from capillary, friction, viscous, and even suction forces [90].

Animals with hairy, or “fibrillar”, adhesive organs include flies [97], beetles (Figure 6B) [94,98], spiders (Figure 6C) [99,100], skinks [8,101], anoles, and geckos (Figure 6D) [5,102,103]. Evidence indicates that fibrillar adhesive organs evolved independently for many of the animals that possess them, including lizard species of skinks, anoles, and geckos [8]. The gecko toe pads are particularly exalted among dry adhesive researchers, due to their impressive performance and, unlike many insect species, do not appear to require capillary forces to generate large interfacial adhesion [7], though evidence has been presented suggesting it may provide an enhancement under some circumstances [43]. There is good reason for the gecko to evolve superior performance: it has unusually large body mass for a climbing animal which relies on adhesion. As length *L* of a creature increases, its volume and therefore mass will increase as *L*^3^, but the area of its adhesive organs increase as *L*^2^. The result is that either their adhesive organs must become disproportionally larger (Figure 6E) or their intrinsic performance per unit area must increase to provide the same secure grip [96]. For this reason, the adhesion strategies employed by larger insects and lizards such as the gecko are especially interesting to researchers interested in developing practical macroscale dry adhesives.

The hairs or fibers of biological adhesive pads are collectively referred to as setae, and among their many benefits is their ability to improve conformation to rough, natural surfaces. Surface roughness varies considerably between surfaces from the nanometer to millimeter scales, and usually includes variations across a wide range of length scales even for a single material or surface type. While smooth and compliant pads can conform well to relatively large variations on a similar length scale as their own, elastic stresses prevent a continuous pad from deforming and flowing around small asperities, thus reducing real contact area for many adherends and, correspondingly, the adhesive strength. Animals, needing to climb on surfaces with all possible length scales of roughness, must possess adhesive organs that adapt appropriately. Fibrillar structures are able to bend and buckle to reach the microscopic pits and valleys that a flat surface cannot reach, effectively presenting a more compliant surface to the adherend [104]. Longer, more flexible fibers present a softer and more compliant interface and are thus more effective for conforming and adhering to the rough surfaces most often found in nature [7].

A positive correlation between the areal density of setae and body mass has been discovered by researchers across a range of six orders of magnitude of body mass, strongly suggesting that smaller, denser contacts can lead to enhanced performance [50]. This observation agrees well with predicted benefits from contact mechanics models, a concept referred to as contact splitting. Creating very small contact points, while maintaining adequate fiber length, presents a challenge as high aspect ratio hairs are susceptible to various forms of damage including stiction, entanglement, and fiber rupture through excessive elongation. Animals requiring very small contact points, such as the gecko, have resolved this problem, in part, through the use of hierarchy; a relatively thick hair or bundle of fibers splits and gives way to finer hairs, finally terminating in nano-scale spatulae which form the final attachment surface [1,7,43,105]. Durability is further enhanced by forming the setae of relatively rigid material, allowing longer and more slender fibers. Gecko setae are formed of keratin, a relatively tough and rigid material with an elastic modulus of approximately 2.5 GPa common to many biological systems including human hair and finger nails [44,106,107,108]. The combination of material rigidity and hierarchy enables gecko setae to reach lengths in excess of 100 µm while having terminal contact points in the form of spatulae only 200 nm wide [8]. Even these long fibers are inadequate to conform to rough and rounded surfaces alone; geckos and other animals employing relatively large adhesive pads instead enjoy a complex deformable sub-structure underlying the thin fibrillar surface which adapts to roughness at larger length scales in addition to performing the complex motions involved in locomotion.

The structure and material properties of setae also play a role in their ability to remain free of contaminants. Cleanliness is essential for any dry adhesive system, since a buildup of particles on the adhesive surface can seriously impede contact to the adherend. Animals relying on adhesive attachment must therefore have methods for avoiding particulate buildup, regardless of attachment method [20]. The gecko’s spatulae have paradoxically been shown to shed particles, and thus avoid contaminant buildup, despite their strong adhesion [7,18]. This occurs in dry conditions, i.e., without the need for water or other fluid to carry the contaminants away from the setae. Though not fully understood, it appears evident that the material and nanostructure of the setae are optimized to ensure that particles will tend to adhere more strongly to the surfaces the gecko walks on than to their setal surface, a process referred to as self-cleaning [18]. The need to self-clean is also likely the primary reason setal structures such as those of the gecko are typically made of very low surface-energy materials, despite the fact that high surface-energy fibers would provide enhanced performance in the absence of cleanliness and durability considerations.

In addition to providing a stable attachment point, an animal’s adhesive pads must be capable of easy and rapid detachment from their adhered surface for effective locomotion. The method of effecting this change, or reversibility, of adhesion in animals is generally one of mechanical manipulation of their limbs and attachment organs. Many insects with smooth pads (arolia) have passive and active methods of folding and unfolding each arolium as they walk, either preventing or initiating peel failure at the interface as necessary [42]. Animals of many size scales with widely varying adhesive morphologies and mechanisms alter their adhesion substantially by controlling the direction of surface shear they apply; attachment is maximized by pulling adhesive pads towards the body, while detachment occurs in the opposite direction [3,42,108,109]. Of these, we will again use geckos as an important and oft-cited example in which directionality is a result of the mechanics of their setal microstructure [5,7,44,108]. Gecko setal arrays extend from their substructure at an angle, a feature which strongly affects their adhesive characteristics. The tilted angle presents a more compliant surface, with fibers that bend in predictable directions rather than chaotically buckle, helping to prevent entanglement and interference between setae even under significant and varying deformation while conforming to rough natural surfaces. The setae are engaged with a surface through a short proximal dragging motion, in which the orientation is of critical importance [108]. Once engaged, the angle a gecko setal array is loaded has a dramatic effect on its adhesive strength, with detachment reliably occurring near a critical angle [44,108]. Measurement of the setal work of adhesion has found that for a range of distal motion angles associated with adhesive detachment the work necessary becomes negative, indicating a net return of energy in contrast to the large dissipation normally associated with a strong adhesive [5]. The complex supporting structure and coordination of the gecko’s motions enable it to take full advantage of the directional benefits afforded by its setal microstructure, able to attach and detach their adhesive toes in milliseconds while running vertically on nearly any surface at speeds comparable to terrestrial animals running on level ground.

#### 2.3.2. Biomimetic Artificial Fibrillar Dry Adhesives

The study and development of artificial dry adhesive systems has overwhelmingly involved designs incorporating fibrillar structures. There is good reason for this, as the most celebrated natural dry adhesives utilize complex arrangements of fibers. To date, despite substantial effort and variety in approach, researchers have yet to produce an artificial adhesive which could fairly be called a practical fibrillar adhesive on par with a gecko’s performance in terms of adhesion, reversibility, and durability. The fibrillar prototypes produced and associated performance testing have nonetheless advanced our collective understanding of dry adhesion and provided adhesives which do in fact out-perform geckos and other natural systems in certain circumstances, though usually at small (<1 mm) length scales. In this section, we discuss the expected benefits of artificial fibrillar structures and give examples of surfaces developed to investigate and exploit these benefits.

Solid surfaces that are atomically smooth are essentially non-existent in the world around us. Instead, all surfaces have some degree of roughness that inhibits adhesive contact between solids, and a successful dry adhesive must account for this in its design as explained in Section 2.2.2. As observed from biological systems, an adhesive surface coated with long and slender fibers is able to conform more easily to surfaces with roughness on a similar length scale as the fibers may bend and buckle as necessary to reduce the force and elastic energy required compared with a smooth adhesive surface. Thus, the enhanced compliance can improve adhesion both by increasing contact area to rough adherends and by increasing the critical energy release rate of the interface. Reducing the effective fiber modulus by increasing its length, for example, was determined in one recent study to increase the inelastic energy dissipation more effectively thus improving the interfacial work of adhesion [110].

Researchers have identified that the shape of a fiber’s tip can affect the adhesion substantially [111], and furthermore that flared or mushroom-like fibers provide superior adhesive strength over competing designs. The performance enhancement of mushroom fibers is most readily explained by the fact that as the fiber is pulled in tension, stresses near the outside edge are reduced due to the greater compliance of the thin spatular membrane, which deforms rather than forming stress concentrations at the interface. The enhanced ability to deform in response to loads near the outer edge reduces stress concentrations, and thus stress is more evenly distributed across the interface compared with flat punch contacts. Fibrillar dry adhesive researchers have consequently focused substantial effort on creating designs which incorporate mushroom fibers [49,112,113,114,115], or the closely related film-terminated fibers which are frequently described as crack-trapping [76,77,79,80,81].

**Figure 7:**
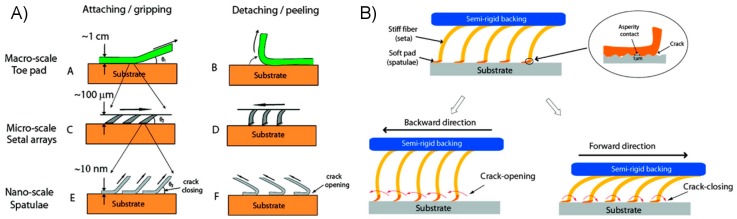
(**A**) Illustrations of gecko feet articulations at the foot, seta, and spatular levels. (**B**) Schematic and actuation of a gecko-inspired synthetic adhesive surface with directional adhesion. (Figures reproduced with permission from [122]; published by ACS, 2009).

Fibrillar designs do not inherently confer reversibility to an adhesive surface. Rather, one must include specific design features to enable this quality. A common strategy directly mimics the adhesive pads of geckos by tilting the fiber, such that adhesion is maximized by shearing in one direction, and detachment occurs in the opposite direction as depicted in Figure 7. Several examples of polymeric fibrillar dry adhesives utilizing tilted fibers have been developed by researchers, indicating adhesive reversibility of up to a factor of 10 [112,115,116,117]. The observation that gecko setae engage with a surface through a short drag distance has inspired the development of adhesives with microfabricated wedge-shaped features [118,119]. Arrays of microwedges were shown to successfully mimic some important aspects of gecko setae, including directionalilty, an impressive load-to-preload ratio, the ability to quickly and easily detach upon the removal of an engaging shear load, and resistance to damage, maintaining at least 67% of their initial performance after 30,000 cycles. Considering the direct influence of gecko observations, it is no surprise that this set of attributes appears specifically well suited for use in climbing robotics [120]. As an alternative to the directionally-dependent adhesion of angled fibers, researchers have additionally created fibers using stimuli-responsive materials in order to reverse adhesion on demand. A thermally sensitive fibrillar array composed of shape memory polymer was used as a structural layer for a thin tacky polymer film, possessing adequate compliance to adhere well to glass surfaces and showing a significant change to reversibility by simply altering the adhesive’s temperature [121].

**Figure 8:**
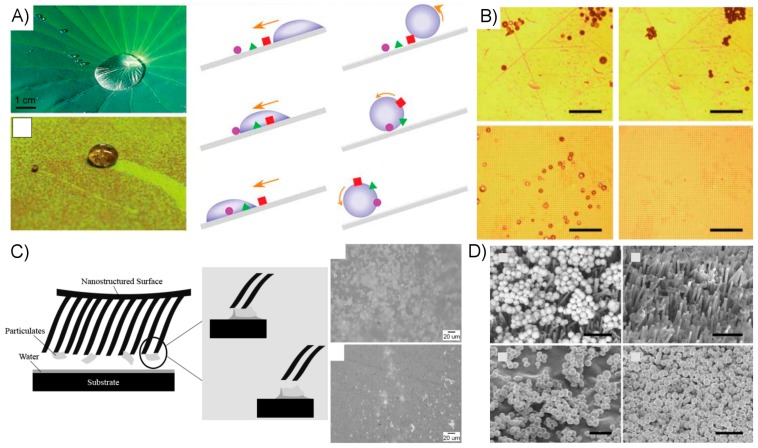
(**A**) Photographs and illustrations of the benefits of hydrophobicity for the removal of surface particles by water droplets (reproduced with permission from [21]; published by Cambridge University Press, 2008). (**B**) Comparison images of a flat (top) and artificial microfiber (bottom) polyurethane surfaces contaminated with silica spheres before (left) and after (right) rinsing with water, showing self-cleaning properties of the fibrillar adhesive (reproduced with permission from [22]; published by ACS, 2009). (**C**) A proposed method of self-cleaning, whereby a thin surface layer of water pulls particles from a hydrophobic nanostructured adhesive surface (reproduced with permission from [124]; published by Wiley, 2007). (**D**) Images of a polypropylene fibrillar adhesive (top) and pressure sensitive adhesive (PSA) (bottom) first contaminated with gold microspheres and then after 30 simulated steps on a clean glass substrate (reproduced with permission from [123]; published by ACS, 2008).

Unlike dry adhesives found in biological systems, many biomimetic fibrillar designs are composed of relatively soft polymeric materials including polyurethane, poly(urethane acrylate) and polydimethylsiloxane (PDMS). The low elastic modulus of these materials enhances tack, and thus improves their ability to adhere to substrates, but also increases their susceptibility to particulate fouling. In most cases, this issue is outside the scope of the researchers’ efforts and thus is given little or no attention. It is however a fundamental concern for the development of a truly robust and practical dry adhesive, and thus some researchers have given the issue special attention. Generally, the hydrophobicity of the surface is cited as an important factor in whether a surface is proficient in the shedding of particles in either wet or dry conditions [25,98,99]. Hydrophobic surfaces are generally made of low-surface energy materials and may be made superhydrophobic through careful use of nanoscale structuring. Figure 8A–C provides examples of natural and artificial surfaces for which the benefits of hydrophobicity for self-cleaning are demonstrated. Figure 8D depicts an example of self-cleaning of a fibrillar adhesive in dry conditions. In this case, the researchers explain that for particles in a certain size range, the fibers make little contact with the particles and surface forces as predicted by the JKR theory are adequate to remove the particles from the fibrillar surface [123].

**Figure 9:**
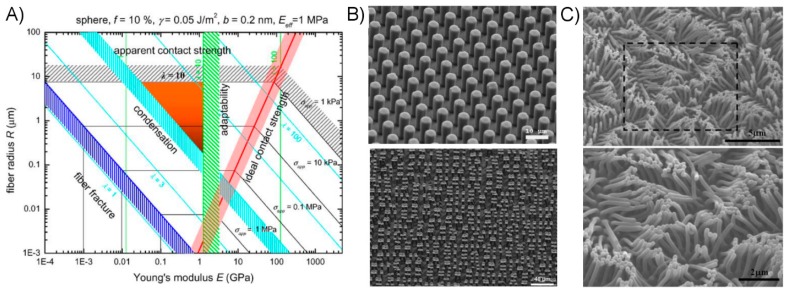
(**A**) Adhesion map for a fibrillar adhesive with spherical tips, given the parameters labeled along the top of the map (reproduced with permission from [125]; published by Elsevier, 2005). Arrays of synthetic pillars showing (**B**) mild condensation by tip contact (reproduced with permission from [127]; published by ACS, 2007), and (**C**) more severe condensation for higher aspect-ratio fibers (reproduced with permission from [128]; published by Springer, 2007).

There is substantial evidence that adhesive performance correlates positively with increased fiber density and correspondingly smaller fiber contact points. Researchers face significant challenges when attempting to create dry adhesives utilizing very slender fibers however, due to both the inherent difficulties in manufacture and fundamental physical limitations that occur as fibers as scaled down in size. The theoretical limits of fibrillar structures are well-studied in the form of “adhesion design maps” [125,126]. These maps are based upon a mathematical description of the various forms of failure which high-aspect ratio fibers may fail (fiber fracture and condensation), compared against the fibers’ requirement to form adequate surface contact (contact adaptability). A given adhesion map is produced given a particular set of assumptions regarding the properties of the interface and adhesive material, and provides a parameter space for an effective fibrillar adhesive system. An example map is provided in Figure 9A for fibers with spherical tips. In general, fiber fracture is expected to become problematic as fiber size decreases and material compliance increases, leading to greater elongation during loading. Fiber condensation, or the sticking-together of neighboring fibers to form a tangle or mat, is an issue inherent in particularly slender fiber arrays and have been often experimentally observed by researchers (Figure 9B,C) [110,127]. Dimensionally smaller fibers created from stiffer materials may enhance adhesion while avoiding condensation related issues assuming contact adaptability remains satisfied.

#### 2.3.3. Alternative Strategies for Enhancing Artificial Dry Adhesive Performance

The difficulties of creating effective fibrillar designs have, in part, inspired work on alternative designs and methods which may prove to be more easily scalable. The alternative designs described herein may be grouped into one of two categories: those which employ passive compliance control determined by their construction and loading direction, and those featuring active compliance control through the use of stimuli-responsive materials.

The term passive compliance control is used here to describe adhesives which are designed such that the compliance changed substantially depending on the direction of loading. The compliance should be large in the direction necessary for conformal bonding to an adherend, but relatively small in the direction of loading to effectively control elastic energy storage and release, similarly to many tilted fibrillar structures. A method of accomplishing this feat is by fabricating an elastomeric composite comprised of a thin, compliant polymer sheet with a relatively inextensible fabric within [58]. The thin and compliant polymer may conform to curved or bumpy surfaces, while the embedded fabric prevents excessive deformation of the polymer when loaded, reducing stress concentrations and enhancing effective adhesive strength. Selecting the point of loading is additionally important; the researchers discovered loading the adhesive sheet from its center via a “tendon” improves adhesive strength in tension, in much the same way that a spatula or “mushroom” cap enhances fibrillar adhesion. Removal of the dry adhesive is easily performed by peeling from its edge, such that the fabric does little to limit the concentration of stress. Multiple recent studies have continued developing this method of creating a practical, scalable dry adhesive. Issues addressed include adhesion enhancement through surface patterning [129], the effect of surface roughness on adhesive performance [62], the importance of the composite properties and geometry [60], and environmental sustainability [61]. A structurally distinct adhesive system for climbing robotic applications has been developed making use of strikingly similar mechanical principles [120]. Several smaller, but more rigid, panels are coated with a microstructured adhesive layer and supported by a compliant structure, with load transferred from each panel to its supporting arm via an inextensible tendon attached to the panel’s center. The effect is a system which behaves as a compliant surface when contacting its adherend, but effectively distributes load across its adhesive interface by virtue of its selectively rigid features. Reversibility is provided by its surface microstructuring, rather than through edge-peeling.

Active compliance control requires a means of actuating, or otherwise stimulating, the adhesive material to alter its mechanical compliance. A relatively simple method of accomplishing this was explored wherein mechanical compression was applied to a polymer film a short distance from its adhesive region with the intent to reduce the system’s overall compliance in the direction of loading [59], showing modest but measurable gains in performance. A composite polymer with magnetic powder was used as a supporting structure in another work [130]. Applying a magnetic field to the composite induced strains which affected compliance according to the orientation of the field. Though this example produced only a minor effect on adhesive performance, magnetic actuation of a material has a strong advantage over many competing methods of active compliance control in terms of speed and the ability to place the energy delivery device relatively far from the active material.

**Figure 10:**
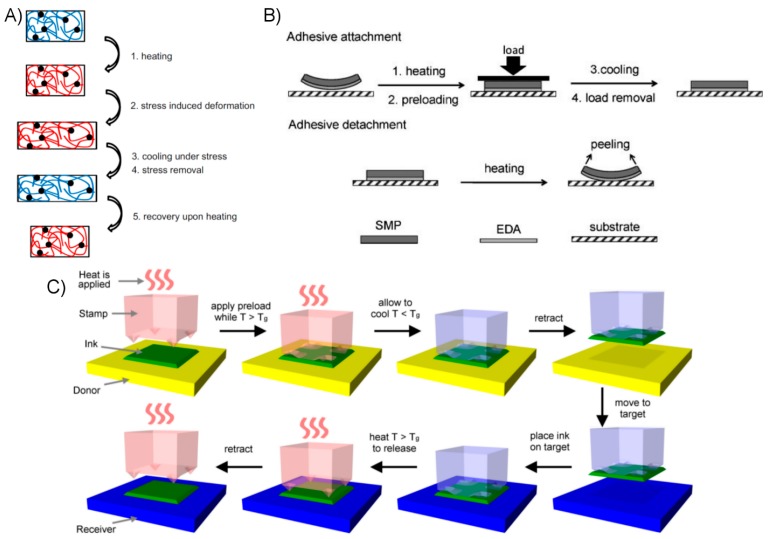
(**A**) The molecular mechanisms of the polymeric shape memory effect, for a thermo-sensitive shape memory polymer (SMP) (reproduced with permission from [131]; published by Elsevier, 2011). (**B**) The bonding and debonding process for a bilayer SMP adhesive (reproduced with permission from [135]; published by Wiley, 2010). (**C**) The transfer printing process of a microscale solid ink using an SMP stamp (reproduced with permission from [136]; published by IEEE, 2016).

A robust and attractive method for compliance control comes in the form of stimulus-responsive polymeric materials, referred to as shape memory polymers (SMPs). An SMP is one which has an original “permanent” shape, which may then be deformed and under certain conditions, fixed into a “temporary” shape, as shown in Figure 10A. Under the influence of a stimulus, usually by heating, the elastic stresses fixed within the deformed polymer are released and it returns to its permanent shape. Over the past decades, researchers have developed many varieties of SMP, with widely varying mechanical properties and mechanisms for producing their shape memory effect [131,132,133]. Chemically crosslinked SMPs that function by undergoing a thermal glass transition are generally the most desirable for dry adhesive applications, due to their chemical, thermal, and mechanical stability. These SMPs tend to be especially rigid below their glass transition temperature (*T_g_*), but soften substantially when heated, changing their compliance by a factor of one hundred or more. The capability of dynamically controlling the compliance, and fixing or “freezing” imposed strains in place to temporarily remove restoring forces, are substantial advantages in terms of allowing the dry adhesive system to control its mechanical behavior. A typical bond/de-bond cycle for an SMP-based dry adhesive involves:

1. A bond phase, wherein the SMP is heated above its *T_g_* to increase its compliance, allowing thorough conformation to the opposing substrate,
2. A cooling and unloading phase, wherein the SMP is cooled below its *T_g_* to reduce compliance and fix its shape, at which point it has maximized its adhesive bond strength,
3. A removal phase, wherein the SMP is re-heated above its *T_g_*, increasing its compliance and releasing stored strains so that it may be removed easily.

Initial examples of using SMP as a component of dry adhesive systems incorporated a separate, “adhesive” layer on the surface, with SMP used as the underlying structural support (Figure 10B). The adhesive layer is chemically similar to the SMP, modified so that its glass transition temperature (*T_g_*) is below the room temperature, and thus the polymer is soft and tacky in normal conditions. Relatively large and simple to produce adhesives using this strategy were developed, where bonding and removal were initiated through heating the SMP above its *T_g_* [134,135]. The reversibility of the adhesives relies on thermal mismatch between the SMP and adhesive polymer to create a “self-peeling” effect when heated. A somewhat more complex design incorporating microscale SMP fibrillar structures terminated with a continuous thin adhesive polymer layer was developed, with the intention of improving the surface compliance during bonding and thus improving adhesion to more rough an uneven surfaces [121].

It is potentially desirable to eliminate the tacky adhesive layer, if in doing so would not prohibitively impact adhesive performance. The surface of an SMP used in dry adhesive applications would be rigid at ambient temperatures and therefore non-tacky and resistant to fouling. In addition, the fabrication process may be simplified somewhat by producing both the structural support and adhesive surface from one unified piece of material. Investigations into this topic have demonstrated that, at least for relatively smooth substrates, a tacky adhesive layer is in fact not necessary to generate strong adhesion. An SMP surface with simple microstructuring was developed, showing impressive adhesive strength [137]. Reversibility was enhanced by the protruding surface microstructures which are compressed during the bonding phase and release their locked-in elastic energy when the SMP is heated for removal. A similar system utilizing these microstructures, termed microtips, has been utilized for small-scale transfer printing where adhesive reversibility is an especially crucial aspect of performance. Transfer printing is, briefly, a field of manufacturing technologies where objects or structures (inks) are fabricated on a donor substrate and then transferred (printed) to a separate receiving substrate [138]. The inks are typically very small or very thin and fragile objects which have little mass, and thus surface forces dominate their interactions with the environment. The transfer is accomplished by precise control of the adhesive forces on their surface, typically using polymeric adhesives, or stamps. The process of printing an ink using a microstructured SMP stamp is depicted in Figure 10C.

## 3. Fabrication

The artificial dry adhesive systems described herein vary substantially in function and composition, and correspondingly require diverse fabrication methods. The method and complexity of fabrication strongly influences the practical size of the adhesive surface. Smaller, higher aspect-ratio surface features with 3D tip shapes and hierarchy have been shown to improve performance in many cases, but add fabrication complexity. Correspondingly, fibrillar structures are generally more challenging to scale up to create large adhesive surfaces than flat or simply-patterned surfaces. Stimuli responsive materials have seen increasing use with dry adhesive systems regardless of surface patterning, imparting enhanced adhesion switchability.

### 3.1. Fibrillar Fabrication Methods and Examples

**Figure 11:**
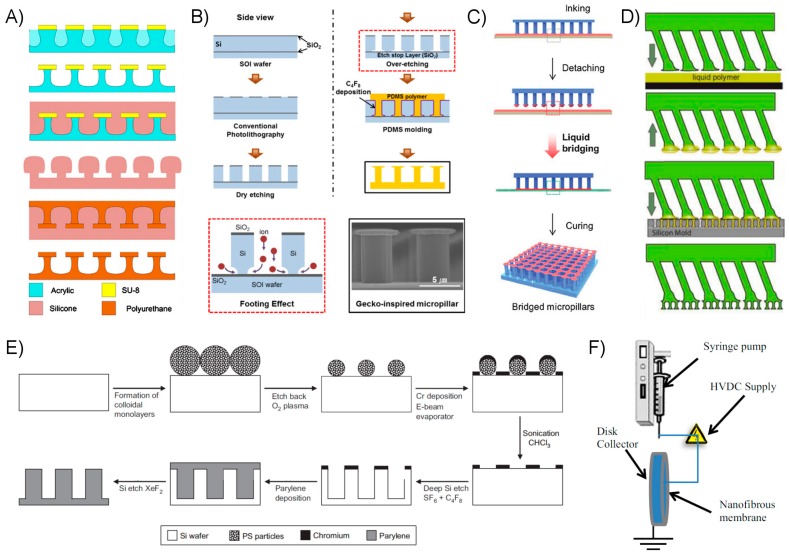
(**A**) Simplified process steps for converting acrylic substrates into mushroom-shaped fibers (reproduced with permission from [142]; published by IOP, 2010). (**B**) The dry etching of a silicon mold for the creation of mushroom-tipped fibrillar adhesives (reproduced with permission from [114]; published by Springer, 2016). (**C**) Using the dip technique to form a terminal film on a fibrillar adhesive surface (reproduced with permission from [81]; published by Royal Society of Chemistry, 2013). (**D**) A process for the fabrication of hierarchical microfibrillar adhesives (reproduced with permission from [105]; published by ACS, 2009). (**E**) The fabrication process using self-assembled nanoparticles as an initial masking layer to define a fibrillar surface (reproduced with permission from [124]; published by Wiley, 2007). (**F**) The method of electrospinning to create nanoscale fibers (reproduced with permission from [147]; published by Springer, 2017).

Microscale fibrillar structures, those with fiber diameters greater than a micron, are most frequently composed of relatively soft polymers such as PDMS or polyurethane (PU) and have features defined by a mold created from traditional photolithographic techniques. A relatively simple approach involves the patterning of a negative-tone photoresist such as SU-8 to create a negative mold. Liquid polymer precursor fills the mold through capillary action, and subsequent curing and demolding give the resulting structure. Variations in this scheme have been used to produce a wide variety of fiber shapes and sizes, and have varying levels of corresponding complexity. Relatively simple vertically-aligned fibers with diameters of a few microns and varying lengths, up to several tens of microns, are straightforward to produce [127]. Producing tilted fibers is accomplished by illuminating the photoresist at an oblique angle when creating the SU-8 mold [112,115]. Using multiple exposures at varying angles, fiber shape may be further influenced to produce wedge-shaped fibers which have broad bases and relatively slender but broad tips [118,139]. A micromachining process to produce a mold with similar wedge features has been demonstrated, where a wedge indentor cuts the features into the mold’s surface [140]. This process is described by its authors as cheaper, faster, and more versatile than the photolithographic method where the resulting wedge quality is highly sensitive to small variations in procedure and equipment quality. Various 3D tip shapes may be produced by dipping the fibrillar polymer surface onto a very thin film of precursor, and then manipulating the cure conditions of the wetted fibers accordingly [112]. Spatula, or “mushroom”, tips may also be created by selectively etching a thin layer of photoresist below the SU-8, creating undercut features [141]. A variation on this approach uses SU-8 as a masking layer for an acrylic substrate which is then etched to produce mushroom-tipped acrylic and SU-8 fibers, from which negative silicone molds may be repeatedly produced (Figure 11A) [142]. The advantage of this process is the larger size and reduced cost of the acrylic substrates versus the silicon generally used with other SU-8 processes.

Despite its cost, etching fibrillar cavities directly into a silicon, or silicon on insulator (SOI) wafer is a common approach due to the ubiquity of silicon processing equipment and technologies. High-aspect ratio features may be produced through deep reactive ion etching (DRIE) of silicon with a suitable masking layer [76,80,81,105,143]. An early example of a synthetic fibrillar adhesive using mushroom or spatula tips which demonstrated enhanced performance over a flat adhesive was created by casting a polymer in a mold produced through DRIE etching of the top silicon layer of an SOI wafer [113]. The buried oxide layer acts as an etching barrier, leading to lateral etching in a thin layer at its surface due to the notching effect from ion scatter (Figure 11B), and is a process since adopted by other researchers [114,144]. An interesting alternative approach involves using a silicon master to hot emboss low-aspect ratio features in a PMMA surface which are then drawn upwards at elevated temperature using an electric field. The fibers flatten and spread by electrowetting upon contact with the opposing electrode to form relatively slender, mushroom tipped fibers [145]. Film-terminated fibrillar surfaces have been created by first producing a micro-fibrillar surface through soft molding of a silicon master, then dipping the fibers in a thin film of polymer precursor to be cured (Figure 11C) [76,77,79,80,81]. Hierarchy may be added through successive molding steps, though this adds considerable complexity and cost to the process (Figure 11D). A three-tiered fibrillar surface with spatula tips at each tier was produced by first creating 3D-printed molds for large stalks, which may be produced with straight or curved structure [105]. Smaller fibers are formed through successive soft molding against photolithographically-produced silicon masters in a process destructive to the silicon molds. Researchers interested in reducing cost and expanding the possible fibrillar materials beyond soft polymers have shown that a variety of fibrillar adhesive structures with complex shapes may be produced directly through 3D laser writing using an acrylic-based negative tone resist [146]. This process was shown to reproduce sub-micron features in hierarchical fibrillar arrays.

The theoretical and practical benefits of creating smaller contact points, and therefore fibers, has been well established. As feature size diminishes below the sub-micron scale into the nanoscale, traditional photolithographic techniques become increasingly challenging and ultimately impractical below diameters of several hundreds of nanometers [116,117], though other creative solutions have been developed and utilized to produce dry adhesives of varying practicality. Fibers in the 100 nm range have been produced from polyimide film with electron beam lithography in a process analogous to traditional photolithographic techniques [148], though fiber density and durability were somewhat limited. A more dense though more irregular forest of similarly sized nanohairs was formed using a self-assembling colloidal monolayer as a mask for chrome deposition on silicon, and ultimately DRIE to form the fibrillar negative mold (Figure 11E). The researchers then deposited a layer of parylene, a hydrophobic and relatively rigid material with an elastic modulus of about 2.8 GPa meant to simulate the material properties of gecko foot hairs. The release process destroys the silicon mold. Similarly stiff fibers made of olefin of a half-micron diameter have been produced from polycarbonate membranes with nanoholes, without the need for special photolithographic techniques [149]. Additional methods include wax indentation [53], and electrospinning (Figure 11F) which can create a soft hierarchical surface, though with fibers in a somewhat unorthodox lateral orientation [150,151]. Germanium (Ge) nanowires coated in parylene have been fabricated by first chemical vapor deposition (CVD) of the Ge followed by parylene deposition, producing a highly hydrophobic and adhesive surface. Carbon nanotubes are frequently used by researchers desiring the smallest possible fiber diameter, capable of creating contact points truly on the scale of nanometers [152,153,154]. The nanotubes are thermally grown at high temperature on a specially prepared surface through chemical vapor deposition, but may be transferred via gluing to microscale fibrillar structures to create a hierarchical structure to further enhance surface compliance [152].

### 3.2. Alternative Approach Fabrication Methods and Examples

**Figure 12:**
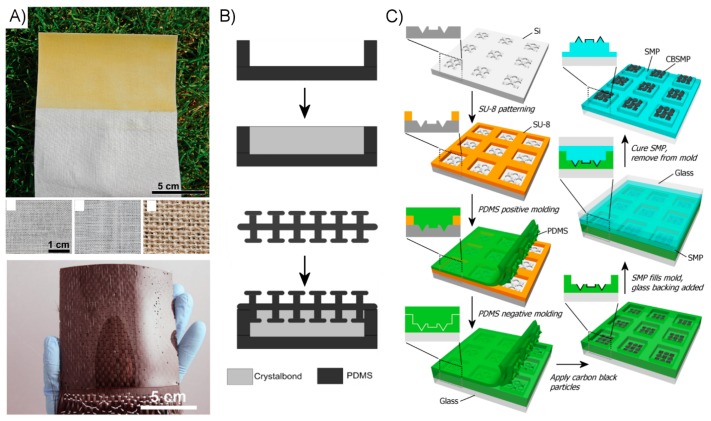
(**A**) Composite adhesives with natural rubber and fabric materials (top) (reproduced with permission from [61]; published by Wiley, 2014), and synthetic polymer with carbon fiber (bottom) (reproduced with permission from [58]; published by Wiley, 2012). (**B**) Fabrication process for dry adhesive samples with an embedded Crystalbond™ phase-changing layer (reproduced with permission from [155]; published by IOP, 2011). (**C**) The photolithographic and soft-molding process for the creation of a composite carbon and SMP laser-activated transfer printing stamp array (reproduced with permission from [156]; published by Wiley, 2016).

Non-fibrillar approaches to creating a dry adhesive generally seek to create a more scalable and lower-cost system. Accordingly, fabrication is generally simpler and results in much larger adhesive surfaces. Fabric and elastomer composite sheets have been created from a variety of natural and synthetic materials in a variety of shapes and sizes (Figure 12A) [58,60,61,62,129]. The production requires little more than preparing a thin layer liquid polymer precursor and carefully inserting a fabric sheet to later be cut to size and fitted with an attachment point for applied loads. An adhesive system utilizing a phase-changing material as a means of dynamically controlling the system compliance was simply fabricated, as shown in Figure 12B by filling an elastomer shell with Crystalbond™ and sealing it.

Large-scale SMP systems are similarly simple to produce. A bi-layer system developed for self-peeling is created with the two dissimilar polymer layers sequentially cured from their liquid precursor form to create the SMP structural layer and the thinner tacky “adhesive” layer [134,135]. An internally-heated SMP system composed of an electrically-conductive SMP layer and a non-conductive contact layer is created in a similar manner [157]. This system may likewise be fabricated in arbitrarily large sheets at low cost and cut to size, with inexpensive copper tape used to quickly form an electrical interface. An SMP surface utilizing microscale surface patterning was created from a re-usable mold requiring basic photolithography and potassium hydroxide etching [137]. Once formed, however, the mold is reusable and could be easily scaled to create surfaces many tens of centimeters in dimension. A similar process is used to produce composite SMP stamp arrays, as shown in Figure 12C. Carbon nanoparticles are embedded within the microfeatures of these stamps to absorb incident laser energy, thus generating the heat required to initiate the shape memory effect and control their surface adhesion, during a transfer printing cycle.

## 4. Performance Metrics and Test Results

The question of how to best measure a dry adhesive’s performance is not a trivial one. In Section 2.1, we discussed several important desirable qualities which a practical dry adhesive should possess, namely controllable adhesion, high adhesion to preload ratio, and durability. Measuring how well a dry adhesive achieves these goals, however, is subject to significant variability between different researchers and different adhesive designs. In this section, the challenges of quantifying and comparing dry adhesive performance are discussed and examples of the most common test methods are provided.

### 4.1. Performance Metrics

The strength of an adhesive bond takes considerable effort to thoroughly quantify. Researchers investigating fundamental dry adhesive physics will generally employ more rigorous testing methods than those primarily reporting novel fabrication techniques, but regardless of the primary motivation all authors seeking to produce useful quantification of their adhesive’s performance face steep challenges. However, the term “strength” can vary remarkably in its interpretation depending upon the type of adhesive, the expected application, and the equipment and techniques available to the researchers. One may broadly classify adhesive strength measurements into ones of normal force, shear force, and work of adhesion. In this case, the work of adhesion should not be confused with the thermodynamic work of adhesion, but rather the actual energy required to separate two surfaces including dissipative effects. However, as alluded to in Section 2.2, the magnitude of force one may expect to measure from a given adhesive interface is strongly influenced by many factors, including the adhesive’s size, apparatus geometry, load distribution, loading rate, ambient temperature and humidity, and the adherend’s material composition, geometry, roughness and cleanliness. It is not unusual for even the same researcher performing identical tests twice to get dramatically different results, and thus quality results demand multiple tests at each condition to give statistically meaningful results.

The presence of normal forces to separate two surfaces is, in most cases, unambiguously caused by adhesive attraction. Most artificial dry adhesives are designed to rely on van der Waals interactions to generate the bulk of this force, although capillary contributions and even mechanical interlocking may play a significant role in some. There are relatively straightforward methods to obtain a quantifiable measure of normal adhesive force at many various length scales, though these different methods should not be assumed to provide directly comparable results. Shear adhesion is somewhat more challenging to measure without applying unwanted interfacial moments and normal forces, subject to the available test apparatus and adhesive geometry. Shear forces at an interface are sometimes described as a friction force, implying that maintaining a compressive normal force is necessary to support the shearing load and also suggesting that the amount of shearing load supported should scale positively with the applied compressive force. In either case, it is frequently shear strength that is of more relevance for dry adhesives used in locomotion both in nature and for artificial robotics. For this reason and others particular to the adhesive geometry and expected application, many dry adhesives are tested exclusively or primarily by quantifying shear force.

Whether reporting shear or normal forces, the reported force is generally the peak force at which uncontrolled failure occurs at the adhesive interface, and is commonly converted to a stress by dividing the force by adhesive area. The conversion of a force to a stress implies a linear relationship between adhesive area and failure load that is nearly always misleading. One can expect with confidence that, for a given set of environmental and loading conditions, adhesive stress as measured in this way will decrease with increasing adhesive area. The source of this relationship is two-fold: due to surface roughness, contaminants, and other defects, the proportion of the adhesive able to make intimate contact with the substrate generally decreases with increasing area, and it becomes increasingly challenging to uniformly distribute the load across the whole of the adhesive interface. Researchers often seek to improve the generality of their results by instead calculating work of adhesion, which when calculated properly will provide a quantifiable value which, in most cases, will better describe the inherent strength and stability of an adhesive interface for comparison across studies. Work of adhesion in this context is a measure of the energy dissipated by separating a unit area of the adhesive/adherend interface, as described in Section 2.2. It is commonly provided in units of J/m^2^ in the case of strong adhesives, and mJ/m^2^ for relatively weaker interfaces. Similar to fracture in solids, where a hard and unyielding material has far lower critical energy release rate than those able to deform and stretch, a tough dry adhesive interface is generally the product of a compliant and deformable adhesive and a strong adhesive may possess a fracture energy of several hundred J/m^2^ against a favorable adherend.

Other measures of performance, notably reversibility and durability, are often given only passing consideration in novel studies. For systems designed with reversibility in mind, the method of initiating adhesive reversal may vary significantly. Fibrillar structures most often rely on an adhesive directionality granted by the fiber orientation. Thus, the reversibility relies on a supporting structure capable of adequate motility to “pull” to adhere and “push” to detach. Even within this class of reversible adhesives, significant differences can exist between the necessary angles and magnitude of shear motion which must be achieved to create the best-case circumstances likely reported. Other researchers will claim reversibility simply by changing the location at which load is applied; an adhesive may offer tremendous shear strength but offer little resistance to peeling if a normal force is applied to one edge. Thus, when reporting and discussing reversibility, one must keep in mind the challenges and individual level of interpretation which may go into a reported value. A recent article promotes the concept of a so-called “adhesion circle” method of measuring directionally variable dry adhesives, to characterize adhesive strength and reversibility in a more thorough and standardized way [158]. Despite the importance of durability for many dry adhesive applications including their use in robotics [15], it is frequently a low priority in fibrillar adhesive studies, which one may rightly suspect is related to the adhesives’ susceptibility to failure. Nonetheless, many researchers make a point of measuring adhesive performance over the course of several adhesive attachment and detachment cycles, though variations exist with regard to the particular source of performance degradation the researchers are measuring. Fibrillar structures in particular are subject to failure through various failure methods outlined in Subsection 2.3.2, but may also be subject to the issue of particulate fouling common to all dry adhesives. Except for adhesives specifically designed with self-cleaning or fouling resistance in mind, adhesive durability is virtually always tested against immaculately clean adherends and thus may provide results which are misleading for real world applications.

### 4.2. Test Methods

The method of testing the strength of a dry adhesive fundamentally involves stressing the interface to the limit at which the interface slips or separates and recording the relevant data. The specifics of the method chosen will depend upon the type and scale of the dry adhesive, the type and thoroughness of data sought by the investigation, and the equipment and expertise available to the researchers. Quantification of the adhesive strength is, in nearly all cases, performed by measuring force in a single linear direction at a time, although repeat tests may be performed to quantify performance for multiple directions relative to the adhesive interface. However, there has been recent work by researchers to develop more sophisticated mechanisms capable of sensing forces in multiple planes simultaneously at length scales and force magnitudes appropriate for many dry adhesive applications, potentially simplifying and enriching the process of data collection [159]. These sensors may be particularly important for the development of robots which utilize dry adhesives, where they are expected to enable real-time feedback to inform the robots’ motion [119]. It is additionally possible to collect valuable information from adhesive tests through the use of high resolution tacile sensors, which provide a two-dimensional map of force versus position across an adhesive interface [160].

**Figure 13:**
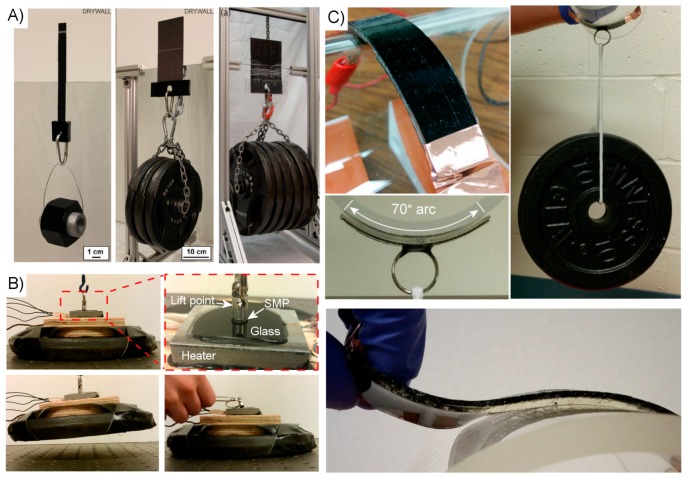
(**A**) Demonstrations of the shear performance of composite polymer adhesives (reproduced with permission from [58,62]; published by Wiley, 2012, 2014). (**B**) A small microstructured SMP dry adhesive demonstrating significant adhesive strength which is reversed by heating (reproduced with permission from [137]; published by ACS, 2013). (**C**) An internally-heated SMP composite demonstrating adhesion to a curved surface which is reversed by heating and peeling (reproduced with permission from [157]; published by MDPI, 2014).

When stressing the adhesive interface, the source of the applied load will generally be either a free-hanging weight, or a motorized or otherwise automated apparatus. Methods employing free-hanging weights are frequently chosen for their advantage of being a very low-cost testing method which is quick to implement, particularly when only a relatively small number of test cycles is needed, or when visual demonstrations are desired such as those shown in Figure 13. Relatively large (>1 cm^2^) or highly adhesive samples are most suitable due to the need for researchers to directly handle the specimens and often to manually apply the weights to the system. Shear and normal adhesion are each relatively simple to measure in this way by orienting the adhesive interface appropriately [154], though most studies report just one or the other. Measurements of SMP-based dry adhesive normal strength have been performed through sequential addition of discrete mass units [157], continuous addition of liquid mass [137]. Adding weight continuously via a liquid pump has the advantage of gradually increasing load at a steady rate, thus improving measurement precision. A number of researchers have chosen to characterize the shear strength of their fibrillar dry adhesives through either directly hanging weights [116,117,123,149,161], or by manual application of force using a spring scale for measurement [153]. A notable shortcoming of these methods is that only measurable quantity of relevance will be the force that occurs at the point of unstable interfacial failure. The lack of electronic force and displacement monitoring precludes more detailed data collection. However, in the absence of a complete set of force and displacement data, work of adhesion may still be possible to estimate using principles of fracture mechanics by making certain measurements or assumptions regarding the crack geometry. An interesting variation of this technique involves rolling a weighted cylinder coated in a dry adhesive surface down a slope, and using its rate of descent to estimate relevant adhesive properties [162].

**Figure 14:**
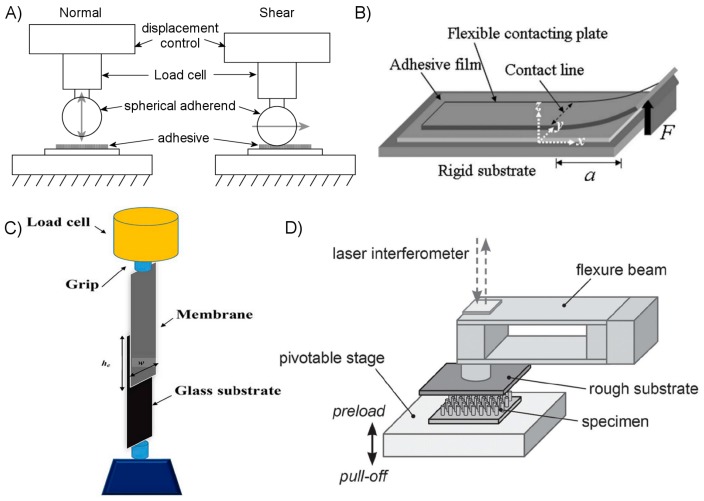
(**A**) A schematic for a displacement-controlled adhesive test with a spherical glass adherend, depicting motion for normal and shear adhesion tests. (**B**) A cantilever method for adhesive measurement (reproduced with permission from [163]; published by APS Physics, 2010). (**C**) A configuration to test adhesive shear force using a tensile tester (reproduced with permission from [151]; published by Wiley, 2017). (**D**) An experimental setup to test adhesive normal force against a flat and rough adherend (reproduced with permission from [167]; published by Wiley, 2016).

Electronically controlled and actuated test apparatuses have a number of appealing features, and are very commonly used for quantitative studies of dry adhesive performance. Test conditions such as preload and loading rate are simple to control with most schemes, allowing researchers to apply a continuously increasing load. Force and displacement data are typically simple to collect with load cells and position sensors respectively, allowing in many cases for a direct calculation of energy dissipation from test data [5,105]. Electronic control allows tests where displacement, rather than force, is controlled (Figure 14A). Displacement controlled tests can offer a variety of investigative benefits for researchers, including the ability to observe crack growth behavior which would be difficult with unstable force controlled methods [74]. Interfacial adhesion is often tested in such setups using a cantilevered arrangement, where the adherend is flexed to allow an interfacial crack to propagate, as shown in Figure 14B [74,163]. Atomic force microscopes (AFM), though not designed with dry adhesive research in mind, are frequently used by dry adhesive researchers in the testing of normal adhesive strength in very small and localized sample regions due to their electronic control and precise force-sensing capabilities. The method is most common with nanofibrillar dry adhesives [124,128,149,164], though it was also notably used to measure the adhesive force of a gecko setae. [44] Similar microscale equipment, such as micro- and nano-indenters, are sometimes used [147,150]. Investigators with much larger adhesive samples often choose material tensile testers, including various tensile testers (Figure 14C) [58,59,60,61,62,129,135,151,165], which like AFMs are pre-existing platforms capable of applying and measuring forces in a repeatable and easily-controlled manner. For those wishing to measure forces for adhesive areas either too large for AFM study, or too small or weak for material tensile testers, the options are somewhat more limited and frequently require the construction of custom test apparatuses. The most commonly used form involves a motorized stage moveable normal to the adhesive surface, and possessing a smooth, spherical or semi-spherical adherend (Figure 14A) [105,111,113,115,127,142,152,166]. Measurements of shearing strength are likewise possible [112,152]. For both shear and normal adhesion experiments, spherical adherends are frequently used to avoid potential mis-alignment issues which may occur with imperfectly oriented flat surfaces, thus intending to improve test consistency. Custom test equipment with flat adherends are nonetheless also used in many investigations of normal adhesive strength (Figure 14D) [49,81,114,144,167].

### 4.3. Test Results

The performance of a selection of dry adhesives as measured by their authors are provided below, organized by the adhesive performance metric used by the authors: Table 1 reports normal adhesion measurements, Table 2 reports shear adhesion measurements, and Table 3 reports work of adhesion measurements.

List of acronyms used in Table 1, Table 2 and Table 3: polycarbonate (PC), polypropylene (PP), polyurethane (PU), poly(ε-caprolactone) (PCL), shape memory polymer (SMP), polydimethylsiloxane (PDMS), carbon nanotube (CNT), single-walled carbon nanotube (SWCNT), multi-walled carbon nanotube (MWCNT), hexamethyldisilazane (HMDS), high density polyethylene (HDPE).

## 5. Applications and Outlook

Nature shows us what is possible in the field of dry adhesion. Dry adhesive research has made great strides in recent years, resulting in artificial adhesives with remarkable strength, a high degree of reversibility, impressive durability and low cost. However, no existing solution yet embodies all of these qualities simultaneously, and even those that claim to satisfy one or more frequently do so only conditionally. Thus, while a long-term goal of artificial dry adhesive research will remain the development of an integrated system conveying the versatility, strength, and longevity of the solutions found in nature, much can be said for the shorter-term development of simpler designs which provide economical solutions for less constrained problems. The adhesive pads and supporting structure of the tokay gecko are indeed impressive, but is mimicking their design the best bet for human applications? One may look to the airfoil for an example where the optimal solution to a problem is not necessarily a direct replication of the one utilized by nature. Biological systems are subject to numerous constraints to which artificial systems are not including the need to be grown and maintained by organic processes, and exist within dirty, wet, otherwise uncontrolled environments where few dry adhesive applications are expected to perform. An adhesive’s ability to adhere firmly to tree bark or wet leaves, for example, is irrelevant for most practical purposes.

The increasingly automated manufacturing facilities of today require non-marring methods of precisely and quickly moving and placing components for device assembly. Dry adhesive methods would appear well suited for this task, and have in fact been the subject of various investigations [81,181,182]. Clean room environments, where delicate wafers are transported from one process site to the next and surface residue must be strictly avoided, are prime candidates to benefit from dry adhesive technology not just for automated handling between processes but also for temporary means of fastening masking layers and other materials to the wafers during processing. This application is a somewhat unique example where the ability to resist fouling is of little consequence, due to the inherent cleanliness required of the operating environment. The set of analogous fabrication techniques, termed transfer printing, likewise operate within very clean environments. At the small scales involved in transfer printing, controlling surface forces through dry adhesion is arguably the only practical method of assembling an expanding selection of heterogeneous structures. Mechanical and electrical bonding of separately-fabricated microscale materials occurs in a process referred to as micro-LEGO, or micro-masonry, where a target micro-object is delivered to its final assembly location via transfer printing and permanently joined through thermal processing [168,183,184,185,186,187,188,189]. The process is capable of producing unique and complex systems which would be prohibitively challenging to create with standard microfabrication. The concept of micro-LEGO can further be adopted to modular electronics and 3D integration, in particular, where the dimension of active and passive devices becomes smaller. The potential value of SMPs to enhance both micro-LEGO throughput and versatility has additionally been recently demonstrated [136,156].

The versatility and reusability of dry adhesives make them well suited for applications in outer space, where suction devices are unusable due to the vacuum environment and non-ferrous spacecraft construction precludes magnetic attachment [190]. Several works have demonstrated dry adhesives which work well in high vacuum and over large temperature fluctuations experienced on the exteriors of spacecraft [190,191,192,193,194], and prototype climbing robots for space applications utilizing dry adhesion have been developed [195]. Progress in dry adhesives is now allowing the technology to be considered for less exotic purposes as well, including the biomedical field where work is being done adapting the technology for wearable sensors [196] and surgical tool grips [197]. Household applications, including reusable wall-hangings and tapes, children’s toys, non-slip surfaces for kitchens, bathrooms, automobiles and more seem likely next steps for dry adhesive technology to take hold. In the meantime, researchers continue working to develop wall-climbing robots which could eventually have a variety of important applications [120,198,199,200,201,202], including helping emergency workers to find and rescue disaster victims, allowing military forces to scout buildings and other locations with greater ease and safety, or by simply giving building maintenance workers a means of inspecting heating ducts and other critical systems. The usefulness of small unmanned aerial vehicles (UAVs) for many of these same tasks is significantly impacted by their typically short flight times. Researchers are working to develop effective dry adhesives which will allow UAVs to perch on walls or other surfaces to conserve energy while observing or awaiting instruction [203,204,205]. A discussion of dry adhesives could hardly be called complete without also considering the possibility of human-scale adhesives allowing us to climb sheer walls with the ease of the aptly-named “Spider-Man”, for which we may happily note that the dream is still alive, albeit with some notable limitations in its current form [16,206]

## Figures and Tables

**Table 1:** Measurements of Normal Adhesion. SMP: shape memory polymer, PDMS: polydimethylsiloxane, PU: polyurethane, PCL: poly(ε-caprolactone), CNT: carbon nanotube, SWCNT: single-walled carbon nanotube, MWCNT: multi-walled carbon nanotube.

Surface Structure	Material	Test Method	Test Scale (mm)	Max. Normal Adhesion (N/cm^2^)	Reversibility	Reference
microtips	epoxy SMP	glass adherend, free-hanging weight	10	200	~1000:1 microstructure, rigidity control	[137]
flat	epoxy SMP	glass adherend, free-hanging weight	100	5–30	-	[157]
microtips, flat, microspheres	epoxy SMP	Si adherend, load cell w/motor stage	0.1	700 (flat) 560 (microtip)	2:1 (flat) ~1000:1 (microtip) microstructure, rigidity control	[136]
microtips, flat	PDMS	Si adherend, load cell w/motor stage	0.1	3–6	>100:1 microstructure	[168]
flat	PDMS	Si adherend, load cell w/motor stage	0.1	7	~100:1 shear displacement	[169]
flat/angled	PDMS	Si adherend, load cell w/motor stage	0.1	10	~100:1 shear displacement	[162]
flat	PU or PDMS/carbon composite	glass adherend, universal mechanical tester, center loading	100	7.5 ^(a)^	300:1 ^(b)^ loading location	[58]
spatula microfibrillar, flat	PDMS Crystalbond filler	6 mm sapphire lens adherend, load cell w/motor stage	1	20	~5:1 fibrillar ~20:1 flat rigidity control	[155]
flat	PCL and bisphnol-A epoxy	Al and stainless steel adherends, universal mechanical tester	10	80 to 650 ~200 solvent self-bonding	>75:1 heat release	[170]
microfibrillar, smooth	epoxy polymer PDMS	4 mm spherical sapphire adherend, interferometer w/motor stage	1	~1 ^(c)^	4:1 loading rate	[171]
film-terminated fibrillar	PDMS	Si adherend, double-cantilever beam	1	2.6 fibrillar ~4 flat	-	[76]
nanofibrillar	CNT	glass adherend, free-hanging weight	4	~10	~10:1 loading direction	[172]
nanofibrillar	SWCNT, MWCNT	glass adherend, laboratory balance	4	12 MWCNT, 28 SWCNT	-	[173]
nanofibrillar	MWCNT	glass adherend, laboratory balance	2	11.7	-	[26]
nanofibrillar	Polyimide	glass adherend, laboratory balance	10	3	-	[148]
microfibrillar	PDMS	Si adherend, displacement sensor w/motor stage	8	0.6 maximum	20:1 shear displacement	[174]
inflatable hemisphere	ST-1060 PU	flat glass adherend, load cell w/motor stage	10	~0.5	204:1 inflation displacement	[175]
gallium liquid	PDMS with gallium liquid	glass, Au, Si, PDMS adherends, load cell w/motor stage	1	2.9 (smooth glass) 3.74 (silicon) 4.4 (gold)	178:1 rough glass 113:1 smooth glass 86:1 silicon Ga phase change	[176]
thick film-terminated fibrillar	PDMS/Fe-PDMS	spherical glass adherend, load cell w/motor stage	10	2.4	minimal, magnetic field orientation	[130]
micro-ridges	PDMS/Fe-PDMS	<1 mm glass sphere, cantilever deflection measurements	1	0.1	~10:1 magnetic field orientation	[177]
microfibrillar, various tips	PU ST-1060, ST-1087	6 mm glass sphere adherend, load cell w/motor stage	6	>0.05	-	[112]
flat	epoxy SMP, elastomer	PC and PP adherends, universal mechanical tester	10	100	>100:1 shape change	[135]
flat, single and dual layer microfibrillar	PU	12 mm spherical glass adherend, load cell w/motor stage	1	2.7 (flat) 5.9 (single layer) 3.75 (dual layer)	-	[105]

^(a)^ Shear (0°) adhesion reported as 29.5 N/cm^2^. From Figure 2b in Ref. [58], normal adhesion is ~25% of this value; ^(b)^ From the caption of supporting information Figure 4 in Ref. [58]; ^(c)^ Estimated from the reported adhesive forces, with 4 mm diameter spherical probe, and reported maximum indentation depth of 100 µm.

**Table 2:** Measurements of Shear Adhesion. PC: polycarbonate, PP: polypropylene, HDPE: high density polyethylene, HMDS: hexamethyldisilazane.

Surface Structure	Material	Test Method	Test Scale (mm)	Max. Shear Adhesion (N/cm^2^)	Reversibility	Reference
nanofibrillar	CNT	Cu adherend, spring scale w/manual force application	10	37 at 25 °C 124 at 1030 °C	-	[153]
nanofibrillar	CNT	glass adherend, free-hanging weight	4	~100	~10:1 loading direction	[172]
nanofibrillar, hierarchical	CNT/SU-8 hierarchical	HMDS-treated 1 mm glass sphere and 1.5 mm roughened steel sphere, load cell w/motor stage	10	~20	large ^(a)^, normal vs. shear loading	[152]
nanofibrillar	SWCNT, MWCNT	glass adherend, laboratory balance	4	7 MWCNT, 17 SWCNT	-	[173]
nanofibrillar	MWCNT	glass adherend, laboratory balance	2	7.7	-	[26]
spatula microfibrillar	conductive PDMS (carbon black)	PP adherend, spring scale	100	0.4	-	[35]
microfibrillar	PP	glass adherend, load cell w/motor stage	20	2	~1000:1 peeling vs. shearing	[178]
microfibrillar, various tips	PU ST-1060, ST-1087	6 mm diameter glass sphere adherend, load cell w/motor stage	1	>0.15	-	[112]
flat	epoxy SMP, elastomer	PC and PP adherends, universal mechanical tester	10	55	>100:1 shape change	[135]
microfibrillar	HDPE	glass adherend, hanging water cup	10	4.7	-	[179]
nanofibrillar	Germanium/Parylene nanowires	self-adhering, wet and dry conditions	5	30	-	[161]
flat	PU or PDMS/carbon composite	glass adherend, universal mechanical tester, center loading	100	29.5 max 26.0 avg.	300:1 loading location	[58]
microfibrillar	PU	6 mm diameter glass sphere adherend, load cell w/motor stage	1	41	-	[180]

^(a)^ The normal adhesion is reported in the supporting information of [152] to be undetectable to the test equipment.

**Table 3:** Measurements of Work of Adhesion.

Surface Structure	Material	Test Method	Test Scale (mm)	Work of Adhesion (J/m^2^)	Reversibility	Reference
film-terminated fibrillar	PDMS	Si adherend, double-cantilever beam	1	0.137 (flat) 1.2 (fibrillar)	-	[76]
nanofibrillar	SWCNT, MWCNT	glass adherend, laboratory balance	4	0.07–0.2	-	[173]
nanofibrillar	MWCNT	glass adherend, laboratory balance	2	0.02–0.08	-	[26]
flat, single and dual layer microfibrillar	PU	12 mm spherical glass adherend, load cell w/motor stage	1	0.002 (flat surface) 0.034 (dual layer)	-	[105]
flat, incised	PDMS	silanized glass plate adherend, cantilever actuated by linear motor w/load cell	10	≤0.8 (crosswise incisions) ~0.1 (smooth surface)	-	[74]
film-terminated fibrillar	PDMS	8 mm diameter spherical glass adherend, load cell w/motor stage	1	0.3 (fiber/film) 0.12 (flat control)	-	[77]
